# Time to Evolve? Potential Evolutionary Responses of Fraser River Sockeye Salmon to Climate Change and Effects on Persistence

**DOI:** 10.1371/journal.pone.0020380

**Published:** 2011-06-28

**Authors:** Thomas E. Reed, Daniel E. Schindler, Merran J. Hague, David A. Patterson, Eli Meir, Robin S. Waples, Scott G. Hinch

**Affiliations:** 1 School of Aquatic and Fishery Sciences, University of Washington, Seattle, Washington, United States of America; 2 National Marine Fisheries Service, Northwest Fisheries Science Center, Seattle, Washington, United States of America; 3 Fisheries and Oceans Canada, Science Branch, Pacific Region, Simon Fraser University School of Resource and Environmental Management, Burnaby, British Columbia, Canada; 4 SimBiotic Software, Ithaca, New York, United States of America; 5 Department of Forest Sciences and Institute for Resources, Environment and Sustainability, University of British Columbia, Vancouver, British Columbia, Canada; Institute of Marine Research, Norway

## Abstract

Evolutionary adaptation affects demographic resilience to climate change but few studies have attempted to project changes in selective pressures or quantify impacts of trait responses on population dynamics and extinction risk. We used a novel individual-based model to explore potential evolutionary changes in migration timing and the consequences for population persistence in sockeye salmon *Oncorhynchus nerka* in the Fraser River, Canada, under scenarios of future climate warming. Adult sockeye salmon are highly sensitive to increases in water temperature during their arduous upriver migration, raising concerns about the fate of these ecologically, culturally, and commercially important fish in a warmer future. Our results suggest that evolution of upriver migration timing could allow these salmon to avoid increasingly frequent stressful temperatures, with the odds of population persistence increasing in proportion to the trait heritability and phenotypic variance. With a simulated 2°C increase in average summer river temperatures by 2100, adult migration timing from the ocean to the river advanced by ∼10 days when the heritability was 0.5, while the risk of quasi-extinction was only 17% of that faced by populations with zero evolutionary potential (i.e., heritability fixed at zero). The rates of evolution required to maintain persistence under simulated scenarios of moderate to rapid warming are plausible based on estimated heritabilities and rates of microevolution of timing traits in salmon and related species, although further empirical work is required to assess potential genetic and ecophysiological constraints on phenological adaptation. These results highlight the benefits to salmon management of maintaining evolutionary potential within populations, in addition to conserving key habitats and minimizing additional stressors where possible, as a means to build resilience to ongoing climate change. More generally, they demonstrate the importance and feasibility of considering evolutionary processes, in addition to ecology and demography, when projecting population responses to environmental change.

## Introduction

Ongoing climate change driven by escalating greenhouse gas emissions threatens to accelerate rates of biodiversity loss with detrimental consequences for ecosystems and humans [1], [2]. Most assessments of extinction risk due to climate change focus purely on ecological or demographic mechanisms affecting species' spatial and temporal distributions [3], [4], [5]; evolutionary processes are rarely considered explicitly [6]. Yet adaptive phenotypic change by means of evolution or phenotypic plasticity can be crucial for population persistence in situations where environmental change leads to altered or novel selection pressures, particularly when demographic rescue from neighboring populations is unlikely [7], [8], [9]. Hence, there is pressing need to understand interactions between evolutionary and ecological processes and the subsequent consequences for the dynamics of natural populations subject to global warming and other forms of environmental change [10].

Here we develop an individual-based model (IBM) to explore (a) potential evolutionary responses of sockeye salmon (*Oncorhynchus nerka*) in the Fraser River, Canada, to changes in river thermal and flow conditions experienced during their spawning migration, and (b) the relative consequences for population persistence under a range of climate change scenarios. Like most aquatic ectotherms, Pacific salmon (*Oncorhynchus* spp.) are highly sensitive to changes in water temperature [11] and their anadromous life cycle subjects them to a range of climate-related challenges in both marine and freshwater environments [12], [13]. Many populations in Canada and the United States have already been lost or are threatened with extirpation, particularly in southern parts of the range where human impacts have been greatest [14]. Climate change is expected to exacerbate population declines in many regions [15], [16], while improving habitat suitability in others.

**Figure 1:**
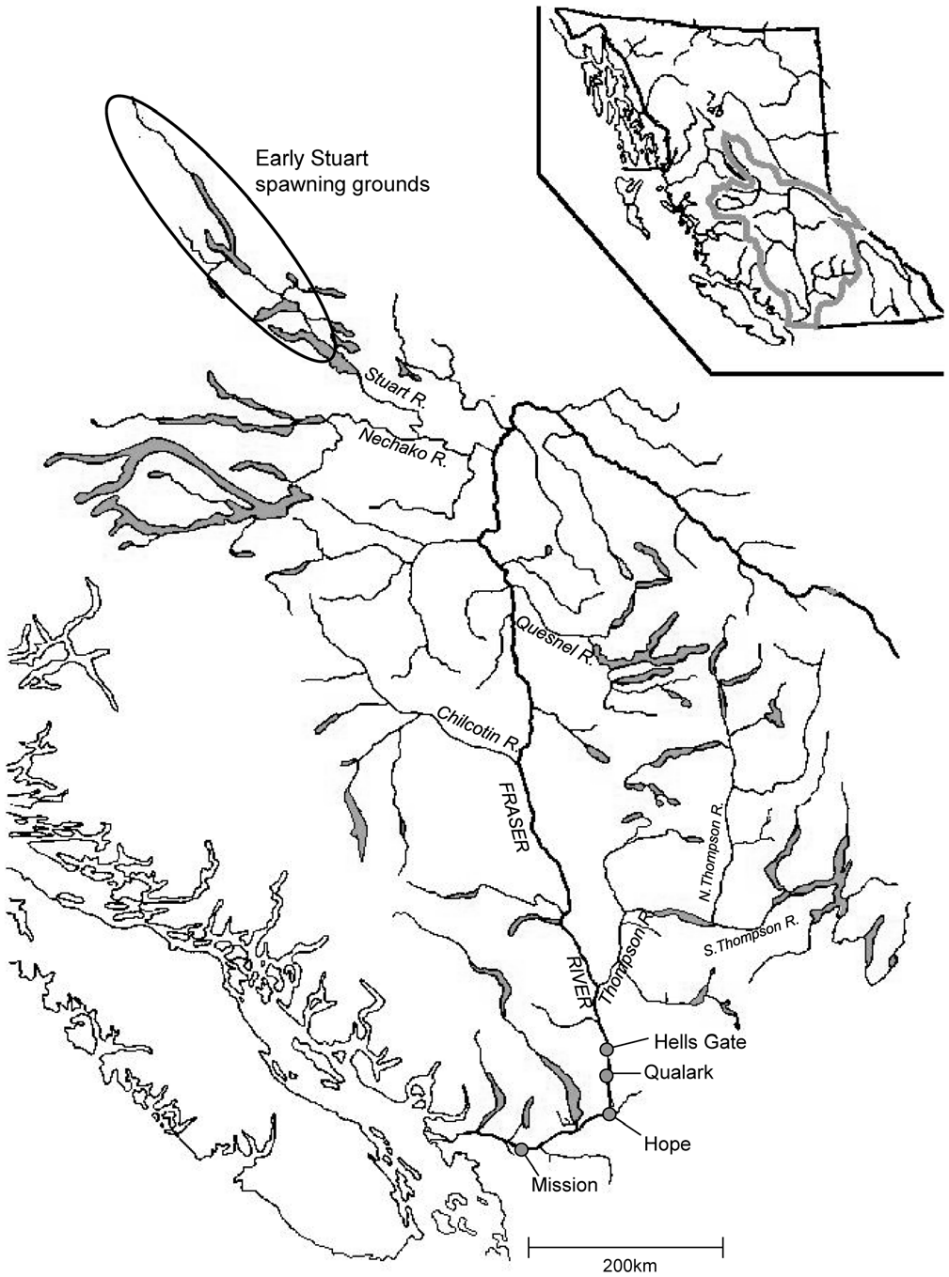
Map of the Fraser River watershed. Map of the Fraser River watershed, with Early Stuart sockeye salmon spawning grounds highlighted with an ellipse. The locations of Hell's Gate, Qualark, and Hope (lower river) are also shown.

**Figure 2:**
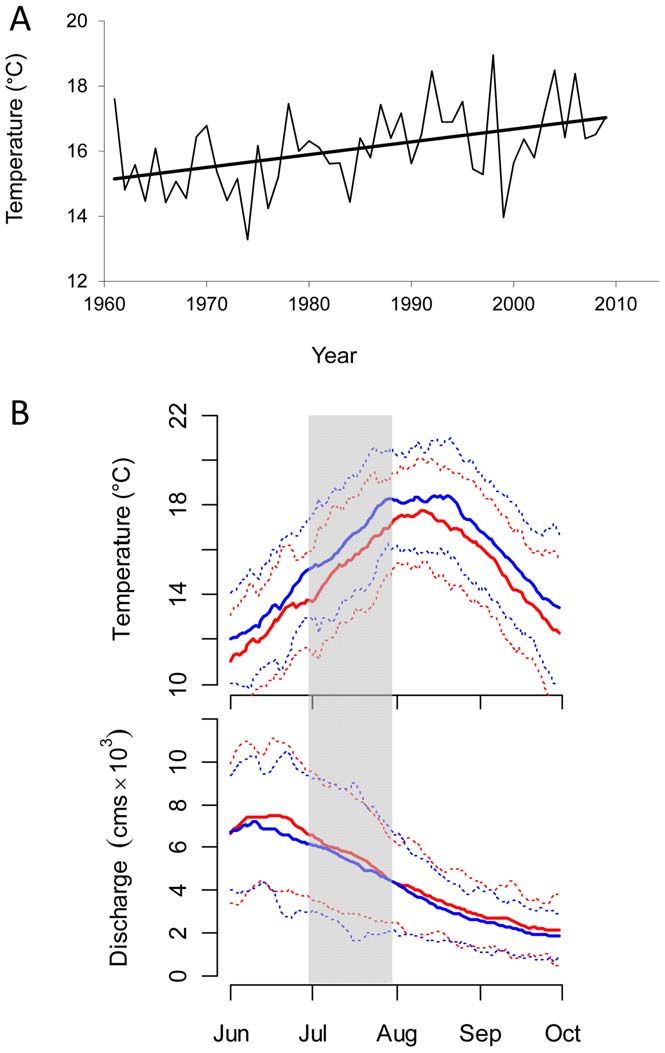
Inter-annual and intra-annual patterns in river temperatures and flows. (A) Average July water temperature at Qualark as a function of year. Dark line indicates significant (*P*<0.01) linear trend towards increasing temperatures (slope = 0.04°C year^−1^). (B) Mean temperature and discharge (±2 SD, dotted lines) for the lower Fraser River for June–October from 1961–1990 (red lines) and from 1991–2009 (blue lines). The shaded area indicates a symmetric 31-day period around the historical median run timing date for Early Stuart sockeye (July 14).

Pacific salmon are anadromous and semelparous; hence, an individual's lifetime fitness is dependent entirely on a single spawning season, which in turn hinges on its ability to successfully migrate from the ocean to spawning sites upriver. The spawning migrations of sockeye salmon in the Fraser River (Fig. 1), where mean summer water temperatures have risen by ∼1.5°C since the 1950s (Fig. 2A; see also [17]), are particularly well-studied (reviewed in [18]). Thermal exposure during upriver migration varies for the numerous spawning stocks in this watershed as a function of their dates of river entry, migration durations and routes, and thermoregulatory behaviors and opportunities [19], [20].

Temperature plays a critical role in mediating many physiological processes affecting fitness in salmon [21]. Energetic costs of migration, susceptibility to disease, rates of disease progression, and stress levels, for example, typically increase at higher temperatures [22], [23]. In sockeye salmon, aerobic scope for performance (defined as the difference between basal and maximal metabolic rates) and cardiorespiratory function are also impaired at high temperatures, limiting migratory ability during thermal extremes [22], [24]. Even if temperatures deviate from those at which aerobic scope is maximal by only a few degrees, increased oxidative stress and pathogen virulence can reduce individual viability [11]. Consequently, expected future increases in summer water temperatures in the Fraser River [19], [25], [26] will increase in-river mortality of migrating sockeye salmon in the absence of adaptive responses, with some stocks predicted to be more affected than others [19], [20], [22], [24]. Increased migratory challenges caused by thermal stress may be partially compensated for by projected decreases in the magnitude of the spring freshet (e.g. due to reduced winter snowpack) [27], although summer-run stocks may experience higher flows if Fraser River hydrology becomes rainfall driven [25].

One potential way for salmon in the Fraser River to avoid suboptimal temperatures or flows is to shift their migration timing. Adult upriver migration timing is thought to be strongly heritable and evolutionarily labile in this species [28], [29], although variation within and across populations is to some degree also associated with direct environmental (rather than genetic) influences [30]. Spawning occurs from late July to December, depending on the population. Currently, to reach their spawning grounds at the appropriate time, some Fraser River sockeye salmon populations migrate before water temperatures in the lower river reach their summer peak (usually early August, see Fig. 2B), while others migrate during, or after, the peak. Future increases in summer water temperatures might select for earlier migration timing in populations that initiate their freshwater migration before the temperature peak and later timing in those populations migrating afterwards [19]. Given the existence of sufficient heritable variation for this behavioral trait and a direct causal link with variation in individual relative fitness [31], populations might be able to evolve different migration timing, effectively reducing overlap with poor migration conditions. However, theoretical considerations suggest a limit to the rate of environmental change that evolving populations can withstand, given that intense viability selection comes with a demographic cost [32], [33]. Whether populations can keep evolutionary pace with a rapidly warming climate remains an open question. Furthermore, some management practices (e.g., supplementation of wild stocks with hatchery fish, habitat alterations, harvest) might affect the diversity of genotypes and phenotypes present in the population, with potentially important consequences for mean fitness [34], [35].

In this study we focus on Early Stuart sockeye salmon, a well-studied stock (i.e., group of populations) that enters the Fraser River in July (Fig. 2B), exposing them to highly variable river temperatures, and also the highest and most variable flows of any Fraser River stock [17], [25]. We used an IBM parameterized generally to reflect the well-characterized population dynamics of lake-rearing, anadromous sockeye salmon [36], but with migration parameters specified using empirical data collected on Early Stuart sockeye salmon. Our goal was to explore relative differences in quasi-extinction risk for a generalized Early Stuart-type life history under a variety of future climate scenarios. We did this by systematically varying key environmental, evolutionary, and demographic parameters to gain a better understanding of how adaptation of migration timing (and constraints on adaptation) might affect future persistence of salmon populations over the next century of warming climate conditions.

## Materials and Methods

### Study system

The Fraser River (Fig. 1) is Canada's largest river discharging to the Pacific Ocean. The hydrograph is driven mainly by snowmelt runoff in spring, with average annual minimum and maximum flows of ca. 620 and 8600 m3/s, respectively (Environment Canada Water Survey of Canada database: http://www.wsc.ec.gc.ca/). Most Fraser River sockeye salmon migrate upstream between late June and late September [17], and for fisheries management purposes are divided into four chronological run-timing groups: Early Stuart, Early Summer, Summer, and Late Run [37].

Early Stuart sockeye salmon are comprised of an aggregate of several demographically distinct spawning populations, which spawn in the far upper reaches of the watershed in the Stuart-Takla Lakes region (Fig. 1). Early Stuart sockeye salmon migrate an extreme distance (1050 to 1200 km) and enter the river earlier than most other Fraser sockeye salmon stocks [median historical river entry date = July 7; 38] before temperatures in the lower river reach their summer peak and just after the peak spring freshet (Fig. 2B). This exposes them to highly variable temperatures in the Fraser River mainstem (interannual mean = 15.8°C, SE = 1.4°C) and also high, variable flows (interannual mean = 5607 m^3^/s, SE = 1406 m^3^/s [17]).

Early Stuart sockeye salmon spawn in small streams associated with Takla and Trembleur Lakes from late July through August. Eggs incubate over winter and fry emerge in spring and migrate to Takla and Trembleur Lakes for rearing. The vast majority of juveniles spend one full year rearing in the lake before migrating to sea as smolts the following spring [36]. After 2 full years feeding and growing in the North Pacific Ocean, mature adults return to the Fraser River in their 4^th^ year of life and migrate upstream to their natal spawning grounds. The majority of Fraser River sockeye salmon exhibit this basic 4-year life cycle, with relatively little interannual variation in age structure, although a small fraction (ca. 10%) spend either an additional year in fresh water or the ocean and thus return to the river in their 5^th^ year [36].

### The effects of river temperature and flow on survival

For sockeye salmon in general, and for those with long migrations in particular, water temperatures above ∼18°C increase *en route* and pre-spawning mortality (PSM) [38], [39], [40], [41], [42]. A recent study by Macdonald et al. [38] showed that when maximum river temperatures experienced by Early Stuart sockeye salmon in the lower Fraser River were high, the ratio of the number of fish estimated on the spawning grounds to the number of fish estimated in the lower river at Mission (Fig. 1) was low, after adjusting for in-river harvest estimates. Both upper and lower river counts were estimated with error, but they did provide an indirect proxy of actual migration survival, consistent with other research. Moreover, these statistical models suggested non-linear effects of high river temperatures on migration survival, with significantly reduced survival associated with average (mainstem) river temperatures above ∼18°C [38]. Studies of other sockeye salmon populations in the Fraser River [19], [20], [41] and the nearby Columbia River [13], [39], [42] have estimated the relationship between migration survival and temperature with more direct methods (e.g., using tagging or telemetry techniques); all have shown that survival typically remains relatively high across a broad range of lower temperatures, but drops off precipitously above temperatures in the 17 to 20°C range, depending on the stock.

**Figure 3:**
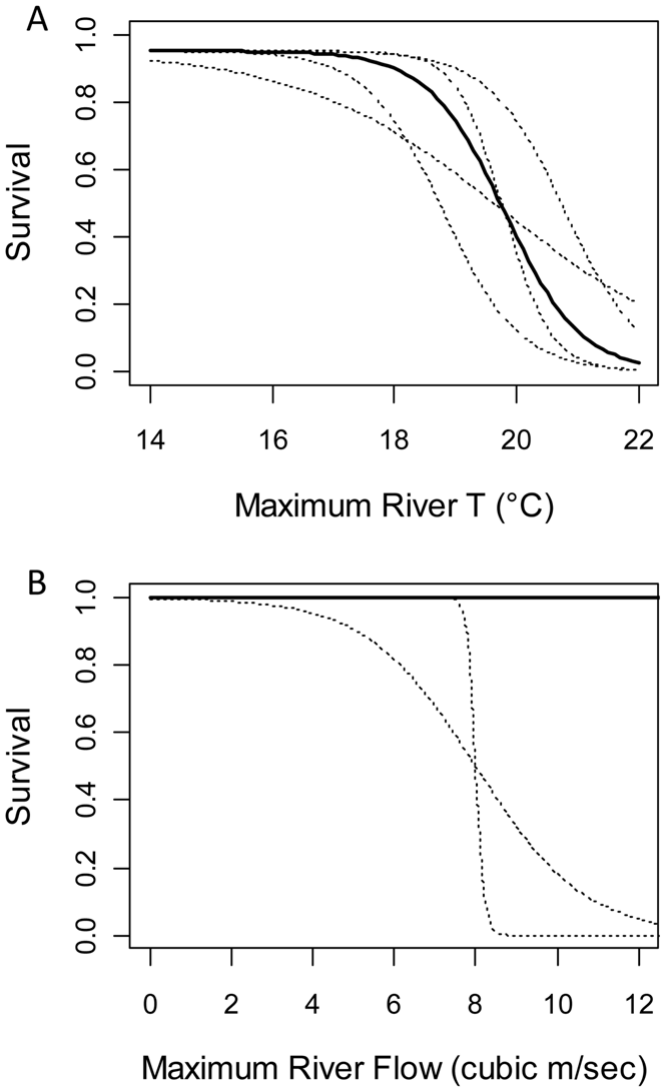
Survival functions used as inputs to simulations. (A) Temperature-survival functions explored. Solid sigmoid curve was used to generate the main results reported in Figs. 4&5. Simulations were also run using the dotted sigmoid curves to explore the sensitivity of the results to the inflection point (*T_50_*) and slope (*b*) parameters of the sigmoid function (results presented in supplementary figure S1). (B) Sigmoid curves used to characterize the relationship between migration survival and maximum river flow. For the main results reported in Figs. 4&5, no effect of flow was modeled (dark line). The sensitivity of these results to including a flow effect was then tested using two alternative survival curves (dashed curves). Note that survival is necessarily zero when there is no river flow (0 m^3^/s), but summer flows close to zero are extremely unlikely to occur in our model.

We used a sigmoid function to reflect the effect of temperature on migration survival:
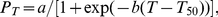
(1)where *P_T_* is the probability of survival at a maximum river temperature of *T*, *a* is maximum survival at cool temperatures, *b* is the rate at which survival declines with temperature, and *T_50_* is the temperature associated with 50% of the maximum survival. The default parameter values used were *a* = 0.95, *b* = 1.6, and *T_50_* = 19.8°C, which produce a sigmoid curve (dark curve in Fig. 3A) that closely approximates the estimated nonlinear effects of high river temperatures on Early Stuart migration survival reported in [38]. Given uncertainty about the exact shape of this relationship for Early Stuart sockeye salmon, we also explored the sensitivity of our results to *T_50_* values of 18.8°C and 20.8°C, and to *b* values of 0.6 and 2.6 in separate simulations (see Fig. 3A, dotted curves).

In addition to the above temperature effects, Macdonald et al. [38] also found that fewer fish apparently made it to the spawning grounds in years where maximum river flows were high (as measured at Hope, BC, in the lower river near Hell's Gate; see Fig. 1). Flows above ∼7000 m^3^/s led to decreases in overall migration survival, while flows >9000 m^3^/s completely impeded sockeye salmon migration [43]. We therefore ran additional simulations where we included an effect of maximum river flow on survival, in addition to the temperature effect. We chose a sigmoidal function to capture the fact that survival remains consistently high at lower flows but drops off significantly at flows >7000 m^3^/s [17]. In the extreme of no flow (or extremely low flows), survival will be close to zero as salmon obviously need water to migrate, but Early Stuart sockeye salmon are not expected to encounter such conditions; thus we only considered values that were high enough to avoid the problems associated with very low flows. The sensitivity of the results to this relationship was also explored using different sigmoidal curves (see Fig. 3B).

### The simulation model

The IBM simulated a closed, but freely-mixing, sexual population, with a 4-yr generation time, fixed age structure, and non-overlapping generations (which provided a good approximation to the life history and age structure of Early Stuart sockeye salmon). The model was not spatially-explicit; rather, individuals were born in a generic ‘river’ environment, migrated from there to a generic ‘ocean’ environment after one full year of freshwater rearing, and then migrated back to the river as mature adults for spawning. Individuals were tracked through an egg phase (from egg deposition in late summer to hatching the following spring, a juvenile phase (1^st^ year) in the river, a sub-adult phase (next 2 years) in the ocean, an adult migration phase (during which mature adults were assumed to be ‘migrating’ in the river to their spawning sites, see below), and finally, a spawner stage. The model ran using weekly timesteps, but events such as hatching or migration were calculated to the fraction of a week, so time was effectively continuous. For simplicity, we assumed a constant, non-selective mortality during the egg phase, and did not include any effects of egg hatching date on juvenile survival. Growth was also not modeled, as we were not interested in growth effects on survival, maturation, or fecundity in this particular application of the model.

Each generation, mean absolute fitness in the population 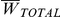 (equivalent to the population growth rate *λ*) had 3 components:
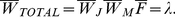
(2)

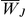
 was the mean survival of juveniles in the river and was density-dependent, 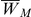 was the mean in-river survival of migrating adults, and 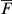 was the mean *per-capita* fecundity. Sockeye salmon females can produce upwards of 2500 eggs [36]; however, to increase the running speed of the model, we reduced 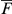 to 5 (separate sexes were not modeled, see below), and adjusted 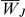 such that a stable population with reasonable year-to-year fluctuations would result in the absence of climate change. Survival at each stage was simulated by drawing a pseudo-random number from a uniform (0,1) distribution and killing the fish if the value was higher than the calculated deterministic survival based on the sigmoid relationships between survival and temperature/flow. This introduces a stochastic element to individual survival (i.e., demographic stochasticity). Juveniles experienced density-dependent survival each generation according to the following stage-specific Beverton-Holt function [44]:
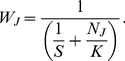
(3)
*W_J_* was survival from the juvenile (fry) to the sub-adult stage, *N_J_* the number of juveniles, *S* the intrinsic survival (survival at very low density), and *K* the carrying capacity of sub-adults. The values of *S* and *K* used in all simulations were 0.7 and 1500, respectively. Survivors subsequently entered the ocean phase, during which survival was assumed, for simplicity, to be 1 for all simulations. The values chosen for the above demographic parameters will obviously affect the resultant population numbers and absolute extinction probabilities in the model. However, our aim was not to create a stock reconstruction of Early Stuart sockeye salmon for comparison with historical time series, nor to project future dynamics in a prescriptive, absolute manner. We were interested in selective mortality at the adult migration phase of the life history and its consequences for relative extinction risk (e.g., for replicate populations with varying degrees of evolutionary potential), so the rest of the life history was parameterized purely to generate population dynamics reasonable for this sockeye salmon ecotype.

In the middle of week 28 of their 4^th^ year of life (equivalent to calendar date July 14, the median historic migration date through Hell's Gate; see below) fish migrated from the ocean back to the river, thereby transitioning to the adult upriver migration stage. An individual's migration timing phenotype *z_i_* (measured in weeks up to 4 decimal places, and expressed relative to the fixed baseline of July 14) was determined by the sum of an inherited additive genetic effect *a_i_*,, equivalent to an individual's ‘genetic merit’ for the trait, and a non-heritable residual effect *e_i_*, which conceptually encompassed non-additive genetic effects, developmental noise and random environmental variation [31]:
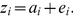
(4)


Hell's Gate in the lower river (Fig. 1) was chosen as a geographic reference point at which to define migration timing and center the impact of river conditions on migration survival. Temperature and flow data have been collected from nearby stations at Qualark Creek and Hope, respectively, for many decades (see below). Early Stuart sockeye salmon typically migrate through Hell's Gate after one week of entering the river mouth at Vancouver, BC. Although fish are potentially vulnerable to stressful temperatures during the entire migration to the spawning grounds (which takes approximately four weeks), we assumed that total migration mortality was directly proportional to the maximum temperatures and flows experienced at Hell's Gate [19], [20], [38]. Migration survival was modeled as the product of *P_T_* and *P_F_*, where *P_T_* was the probability of survival as a function of maximum river temperature experienced at Hell's Gate during the first week of the migration phase (see equation 1 and Fig. 3A), and *P_F_* was the probability of survival as a function of maximum river flow at Hell's Gate (given by the sigmoid survival-flow function, Fig. 3C). *P_F_* was set to 1 for scenarios where we were only interested in the separate effect of temperature.

### Reproduction and inheritance model

Fish surviving the migration became spawners. We used a random-mating, hermaphroditic model without selfing. Families were formed by selecting two parents at random from the *N_A_* surviving adults, who together produced 2*F* offspring, where *F* was the *per-capita* fecundity. For simplicity, *F* was modeled as constant through time and without variation across individuals. Parents were returned to the mating pool and could be selected again for another mating (without possibility of selfing) for a maximum duration of 10 weeks, after which they died. This long spawning period was chosen to ensure that mating was random with respect to migration timing phenotype, rather than to reflect the actual duration of spawning in the wild. We assumed random matings because assortative mating necessarily leads to increasing additive genetic variance in the model, which complicates interpretation of any evolutionary responses or effects on persistence. The expected number of families produced per spawner per week was fixed at 0.05, such that an expected total of 0.5*N_A_* families of offspring would be formed by the time all fish died. Eggs hatched the following spring and immediately became juveniles, thereby restarting the life cycle.

Inheritance rules were based on the infinitesimal model of quantitative genetics theory, which accurately predicts evolutionary responses of polygenic traits to selection over timescales of tens of generations [31]. We assumed a large number of unlinked loci affecting the trait, a Gaussian distribution of *a_i_* values, and constant genetic variance. Offspring inherited their genetic predisposition to migrate upriver at a certain date from their parents. Each offspring's *a_i_* value was drawn from a random normal distribution centered on the arithmetic mean of the two parental values. The variance of this distribution was equal to half the total (population-level) additive genetic variance for the trait, which was an initial input parameter, assumed to remain constant across generations (analogous to the expected genetic variance at linkage equilibrium) [45]. The realized additive genetic variance in any generation could still deviate from the initial additive genetic variance as a result of selection or random sampling of parents. The residual (*e_i_*) component of the trait was drawn from a normal distribution of mean 0 and variance 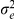. Offspring phenotypes were then formulated according to Eq. 4 above.

The population and evolutionary dynamics were simulated using an IBM developed in collaboration with SimBiotic Software (www.simbio.com), within their SimUText program. Additional details and a user guide to the model are available on request (http://simbio.com/contact).

### Scenarios explored

We examined a range of different climate change scenarios. River temperatures and flows were read into the IBM at weekly timesteps. Baseline profiles were generated by obtaining historical daily river temperature and flow data, measured at Qualark and Hope, BC (Fig. 1), respectively. Flow data are available from the Environment Canada Water Survey of Canada online database (www.ec.gc.ca). Temperature data were provided by the Department of Fisheries and Oceans Canada's (DFO's) Environmental Watch Program [17]. Data were first summarized into weekly averages and then averaged across the period 1990–2009 (see blue lines in Fig. 2B), to produce baseline (i.e., recent historic) seasonal profiles for river temperature and flow (hereafter thermograph and hydrograph, respectively).

For baseline scenarios, we simulated the recent historic thermograph and hydrograph by adding random deviates to existing seasonal patterns, effectively increasing or lowering the entire thermograph/hydrograph each year (i.e., the model added random inter-annual, but not intra-annual, variance to the seasonal profiles). Deviates were generated by drawing two random numbers each year from a bivariate normal distribution of means 0, standard deviation of 1°C for temperature (approximating the observed historic interannual variation in July temperatures at Qualark), standard deviation of 1500 m^3^/s for flow (approximating the historic interannual variance in July flows at Hope) and correlation of −0.75 (reflecting the observed negative correlation between July temperatures and flows).

For scenarios with climate change, we assumed a continuous linear increase in average annual temperatures through time. We examined eight different rates of river warming, from a minimum 0.5°C increase in mean river temperatures by 2100 (starting in 2010 - i.e., a rate of 0.005°C/year) to a maximum 4.0°C increase (i.e., a rate of 0.044°C/year), in increments of 0.5°C. Morrison et al. [25] predicted an increase in average summer water temperatures of 1.9°C by the end of the 21^st^ century for the Fraser River, using output from general circulation models (GCMs) and downscaling methods. Ferrari et al. [26] projected a similar rate of river warming through until 2100, with little apparent differences between months or seasons. Both of these studies applied similar moderate emissions scenarios (CGCM1 and A1B greenhouse gas and sulfate scenarios, respectively) [46]. Realized rates of river warming could be considerably higher for the Fraser River, given recent rates of warming observed since the 1950 s [17], so we also explored increases of up to 4°C.

For flow, we used a time series (years 2010 to 2100) of modeled future flows reported in Morrison et al. [25]. The Morrison et al. model predicts modest changes (∼5%) in average annual flows for the mainstem Fraser River, but significant changes to the seasonal distribution of flow; peak flows are expected to be lower and occur increasingly earlier in spring. While the absolute magnitude of these changes will depend on the realized amount of climate warming, there is considerable uncertainty in the hydrodynamic models used to generate the flow forecasts [19], [25]. Hence, we used the same time-series of modeled future flows regardless of the river warming scenario. The Morrison et al. flow predictions incorporate interannual variation to a certain extent, but this is nonetheless underestimated by their model [25]. To compensate for this, we added extra random variation to future flows (by drawing random flow deviates from a bivariate normal distribution as before, but with a standard deviation of 250 m^3^/s), thereby ensuring that the expected inter-annual variability in future flows matched observed historic variability.

We expected that changes in river temperature and flow during the next century would favor early-migrating Early Stuart individuals. An evolutionary response to this selection pressure would only occur, however, if migration timing is heritable. Narrow-sense heritability (*h^2^*) is defined as the ratio of additive genetic variance in a trait to the total amount of phenotypic variance. The per-generation evolutionary response (i.e., the expected change in the mean trait value) is then given by the product of *h^2^* and the resulting selection differential [31]. A recent review of quantitative genetic studies of salmonids reported a median heritability value of 0.51 for phenological traits across studies [47]. In the absence of direct information on the heritability of migration timing for Early Stuart sockeye salmon, we explored 4 different *h^2^* values for each climate change scenario: 0, 0.25, 0.5, and 0.75, corresponding to increasing evolutionary potential for a given magnitude of phenotypic variance.

A default phenotypic standard deviation (PSD) of one week was specified as an input parameter to the model, approximating the typical within-year spread of migration dates observed for Early Stuart sockeye salmon [48]. When scaled by the heritability, this parameter determines overall evolutionary potential in our model; i.e., the amount of additive genetic variance upon which selection can act in any given generation. Depending on the form and strength of selection, the magnitude of phenotypic variance can also strongly affect the mean fitness of populations experiencing a moving phenotypic optimum [32]. We examined these potential consequences by varying the phenotypic variance in upriver migration timing from a minimum of 0.5 to a maximum of 2, in increments of 0.5. For all simulations, we also included random inter-annual (i.e., general environmental) variance in migration timing by drawing a random deviate from a normal distribution of mean 0 and standard deviation of 0.4 weeks and adding this number to all individual's migration timing phenotypes in that year. This ensured that the resulting interannual variation approximated the observed historic interannual variation in median Hell's Gate peak migration dates [48]. No temporal trend in annual median migration timing (*P*>0.1) or correlation between median migration dates and average July river temperatures (*r* = 0.09; *P*>0.1) was detected in the historical time series (1977–2009), nor was any directional evolutionary change in migration timing predicted by the model in a retrospective analysis (using temperature and flow data from the last 50 years; unpublished results).

Initial population size at the beginning of each model run was 600 adults. For each run, we calculated the realized population mean migration timing each generation by averaging the trait values of live individuals just prior to river entry. Average changes in this metric were then examined across replicate model runs, to assess evolutionary responses. We also assessed the probability of quasi-extinction over the whole run, defined as the proportion of 100 replicate populations where <50 migrating individuals remained by the year 2100.

## Results

### Evolutionary responses to simulated climate change

**Figure 4:**
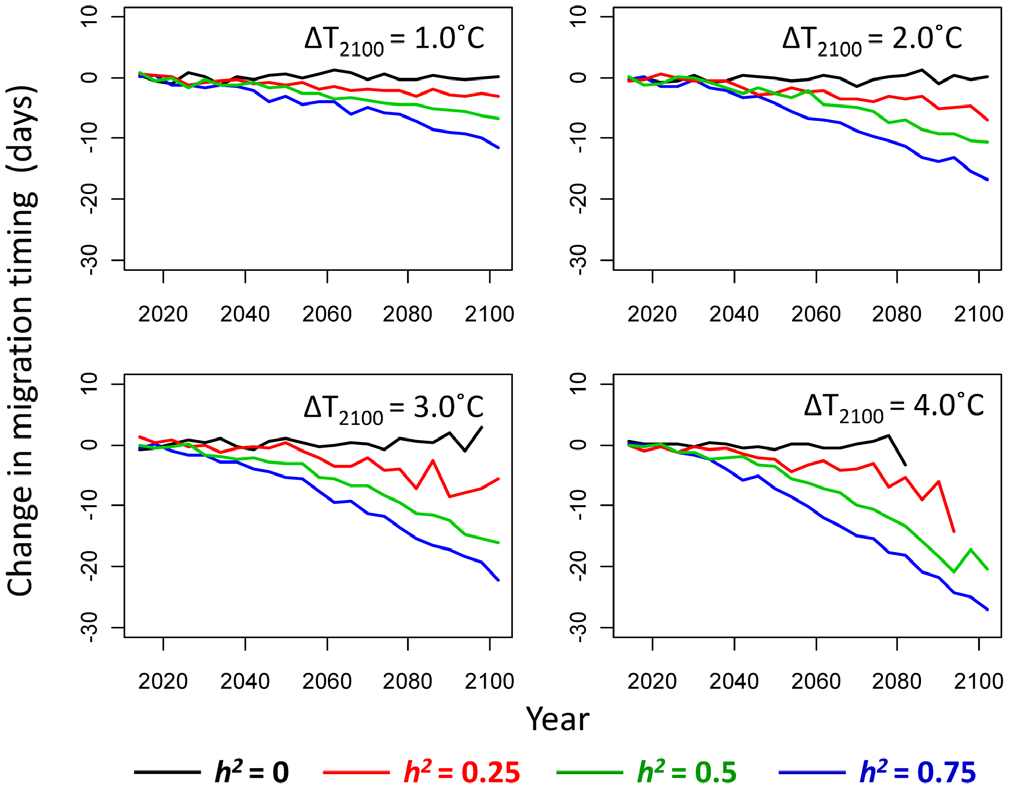
Projected evolutionary trajectories for each warming scenario. Projected future evolutionary changes, averaged across 100 replicate model runs, in mean migration timing (days relative to the historic median Hell's Gate migration date of July 14) for different simulated river warming scenarios, assuming no flow effect on survival (i.e., *P_F_* = 1). Black curves: heritability (*h^2^*) of migration timing = 0; red curves: *h^2^* = 0.25; green curves: *h^2^* = 0.5; blue curves: *h^2^* = 0.75.

Simulated increases in future summer temperatures selected for earlier migration timing and evolutionary responses to this selection pressure occurred whenever heritability was non-zero (Fig. 4). Larger evolutionary responses occurred when the rate of river warming was faster, and for a given rate of warming, rates of evolution were higher for higher heritabilities. With 1°C of river warming by the year 2100, for example, mean migration timing advanced by approximately 7 days when heritability was 0.5 (Fig. 4, top-left panel, green line). The equivalent evolutionary responses were approximately 10 days for scenarios with 2°C of warming (Figure 4, top-right panel) and 13 days for scenarios with 3°C of warming (Figure 4, bottom-left panel), and a heritability of 0.5 in each case. Rates of evolution scaled approximately linearly with the magnitude of heritability in each warming scenario.

With no evolutionary potential (i.e., heritability = 0), populations exhibited no evolutionary change regardless of the warming scenario (Fig. 4, black lines). Mean migration timing in any given replicate populations could still deviate randomly from year to year as a result of random environmental effects or genetic drift. At more extreme rates of climate warming (≥3°C increase by 2100), many replicate populations went extinct and the resulting average evolutionary trajectories became more erratic over time (Fig. 4, lower panels), as persisting populations were reduced to very low size (<100 adults) and therefore more affected by genetic and demographic stochasticity. For all nonzero heritability values explored, the rates of evolution tended to accelerate through time, up to certain point. Once temperatures exceeded ∼18°C, an increasing portion of migrating individuals (in particular, those migrating later) were exposed to stressful temperatures, imposing selection for progressively earlier migration timing. With the default sigmoidal survival function (the solid curve in Fig. 3A), the strength of directional selection increased gradually until temperatures exceeded the inflection point of 19.8°C (i.e., *T_50_* parameter: the temperature at which expected survival is 50% of the maximum), beyond which the rate slowed down.

For simulations where *T_50_* was changed to 18.8°C, stressful temperatures were encountered early in the simulations and as a result, earlier migration timing evolved much sooner (Fig. S1). With this *T_50_*, mean migration timing advanced by almost 20 days for a heritability of 0.5 and a rate of warming of 2°C (Fig. S1; panel A). The equivalent advance was ∼10 days for a default *T_50_* of 19.8°C (Fig. 4, top right panel, green curve). Conversely, when *T_50_* was set to 20.8°C, earlier migration timing evolved only towards the very end of the simulations (Fig. S1, panel C), as stressful temperatures were not encountered at a high frequency until at least the 2060 s. The absolute magnitude of evolutionary response was slightly sensitive to the *b* parameter of the sigmoidal survival function (i.e., the rate at which survival decreased at high temperatures). With a shallow rate of decrease (*b* = 0.6), rates of evolution were slightly slower (Fig. S1, panel E), while with a steeper rate of decrease (*b* = 2.6), rates of evolution were slightly faster (Fig. S1, panel G).

### Effects of evolution on population persistence

**Figure 5:**
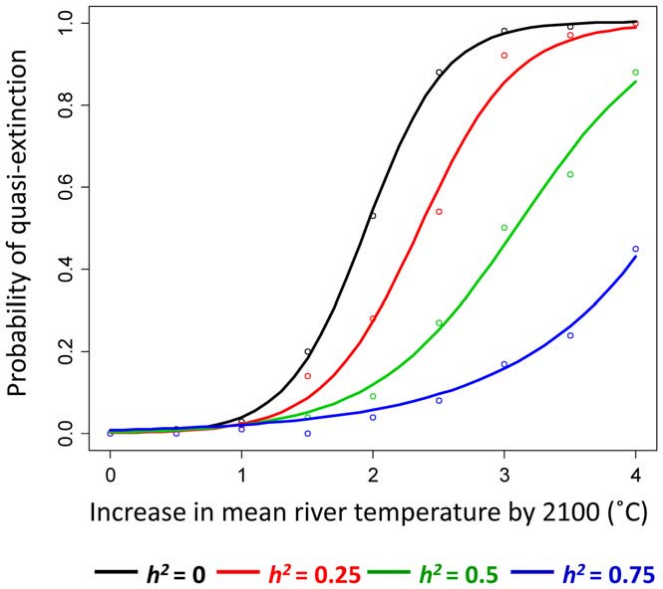
Effects of evolution on quasi-extinction risk. Probability of quasi-extinction as a function of the rate of river warming, assuming no flow effect on survival. Data points show means of 100 replicates; curves are best sigmoid fits to data. Black curve: heritability (*h^2^*) of migration timing = 0; red curves: *h^2^* = 0.25; green curves: *h^2^* = 0.5; blue curves: *h^2^* = 0.75.

Regardless of whether evolution occurred, the probability of quasi-extinction tended to increase approximately as a sigmoidal function of the rate of river warming. However, with zero heritability for migration timing (and therefore no evolutionary responses), the probability of quasi-extinction was much higher for all rates of increase in river temperatures, compared to scenarios where the trait could evolve (Fig. 5). For example, with 2°C of warming by 2100 (and the default sigmoidal survival function), there was a 53% chance of quasi-extinction by 2100 when heritability was zero (black curve in Fig. 5), whereas the equivalent quasi-extinction risk was only 9% when heritability was 0.5 (green curve in Fig. 5). The equivalent quasi-extinction risks for a rate of warming of 4°C were 100% for a heritability of zero and 88% for a heritability of 0.5. Thus, a heritability in the range that has been estimated for phenological traits in salmon reduced the probability of quasi-extinction by more than five-fold under moderate warming, and under an extreme warming scenario the ability to evolve at least provided an opportunity for some populations to persist.

For a given rate of river warming, absolute extinction risk was sensitive to *T_50_* of the underlying sigmoidal survival-temperature function, holding other parameters constant. With a *T_50_* of 18.8°C (i.e., 1°C less than the default *T_50_*), populations with zero heritability had a 40% chance of quasi-extinction even with no directional trend in river temperatures, and a 100% chance of quasi-extinction when river temperatures increased by 2°C by 2100 (Fig. S1, panel B, black curve). In contrast, the equivalent quasi-extinction probabilities when heritability was 0.5 were 0 and 48%, respectively (Fig. S1, panel B, green curve). Thus, the relative effects of evolution (as indexed by heritability) on quasi-extinction probability were similar regardless of the value of *T_50_*: a lower *T_50_* simply shifted the curves to the left (Fig. S1, panel B), while a higher *T_50_* shifted them to the right (Fig. S1, panel D). However, increasing the *b* parameter (steepness) of the sigmoid survival function had stronger effects on relative, compared to absolute, quasi-extinction risk (i.e., the different heritability curves are more widely spaced in panel H of Fig. S1 compared to panel F).

### Effects of future changes in river flow

**Figure 6:**
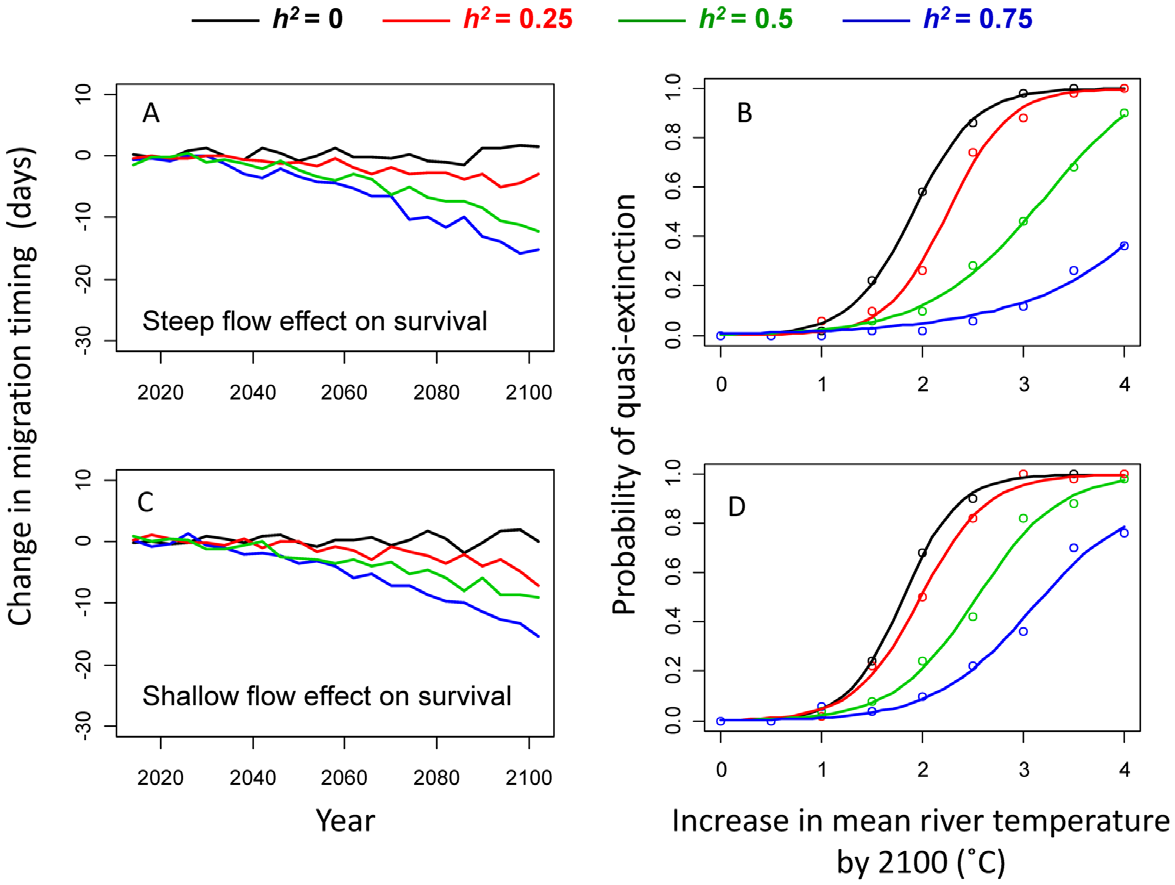
Sensitivity of main results to flow effects. Sensitivity of results to including a flow effect on migration survival. Left panels show projected evolutionary changes in mean migration timing for a scenario where mean river temperature increases by 2°C by 2100, assuming either a steep flow effect (A) or a shallow flow effect (C) on migration survival. Right panels show probability of quasi-extinction across all river warming scenarios for the same steep (B) and shallow (D) flow effects on survival. The default sigmoid temperature-survival curve (dark curve in Fig. 3B) was used in all cases. Data points show means of 100 replicates; curves are best sigmoid fits to data. Black curves: heritability (*h^2^*) of migration timing = 0; red curves: *h^2^* = 0.25; green curves: *h^2^* = 0.5; blue curves: *h^2^* = 0.75.

When migration survival was dependent on both river flow and temperature, the effects on evolutionary trajectories were quantitatively similar compared with those where a flow effect was not included (Fig. 6). With 2°C of river warming by 2100 and a strong threshold effect of flow on survival (i.e., the steep sigmoid dotted curve in Fig. 3B), mean migration timing advanced by ∼11 days when heritability was 0.5 (Fig. 6A, green curve). The equivalent advance in migration timing for a less-steep flow effect (shallow dotted curve in Fig. 3B) was ∼8.7 days (Fig. 6C, green curve), while the advance that occurred when no flow effect was modeled (solid flat line in Fig. 3B) was ∼10.5 days (Fig. 4C, top right panel, green curve).

Similarly, including a flow effect did not make a large difference to the relationship between quasi-extinction risk and rate of river warming that emerged for each heritability treatment, at least for a steep threshold flow effect (Fig. 6B). However, when the survival-flow function was shallower, the relative differences in quasi-extinction risk between heritability treatments were less pronounced for each rate of river warming explored (Fig. 6D).

### Effects of phenotypic variance

**Figure 7:**
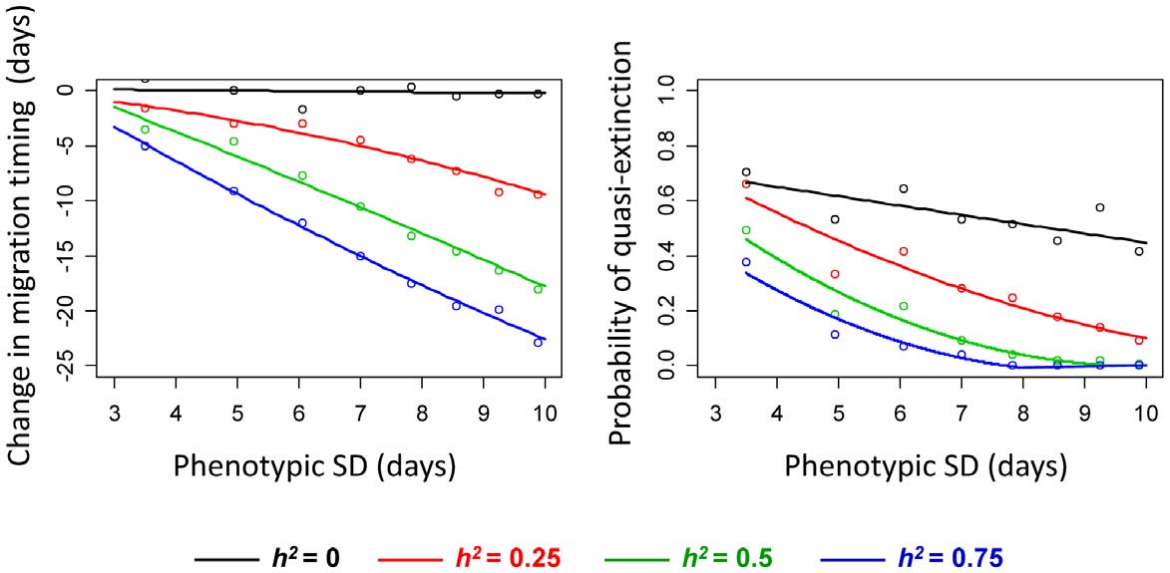
Effect of phenotypic variance on evolutionary trajectories and population persistence. Total change in mean migration timing by 2100 relative to the historic median (A) and probability of quasi-extinction by 2100 (B) as functions of the phenotypic standard deviation in migration timing. Currently, the phenotypic standard deviation in migration timing for Early Stuart sockeye salmon is approximately 7 days. Data points show means of 100 replicates; curves are best linear or quadratic fits to data. Black curves: heritability (*h^2^*) of migration timing = 0; red curves: heritability = 0.25; green curves: heritability = 0.5; blue curves: heritability = 0.75.

The absolute magnitude of evolutionary response (i.e., the change in peak migration timing by 2100, relative to the historical median) for a given rate of river warming was strongly affected by the initial phenotypic variance in migration timing, particularly when heritability was high (Fig. 7, left panel). The probability of quasi-extinction was also lower when the phenotypic variance was higher, and this effect was also stronger at higher heritabilities (Fig. 7, right panel).

## Discussion

Our simulation results show that future climate change is likely to select for earlier upriver migration timing in Early Stuart sockeye salmon, and that evolutionary responses to this selection pressure could substantially increase the probability of population persistence under realistic scenarios of river warming. Forecasts of population trajectories that ignore adaptation are therefore likely to be overly pessimistic. With a simulated 2°C increase in average river temperatures by 2100 (Fig. 4B) and a heritability of 0.5, migration timing advanced by approximately 10 days (1.4 PSD), while the equivalent evolutionary shift for 3°C of river warming (Fig. 4C) was approximately 15.7 days (2.2 PSD). Rates of evolution tended to accelerate over the century as temperatures continually rose, because on average an increasing proportion of individuals were exposed to stressful temperatures (>18°C) each successive generation. Early migrating genotypes were therefore at an increasing selective advantage. For a given rate of river warming, evolution of migration timing resulted in substantially increased population viability, as defined by our quasi-extinction risk metric, relative to situations where migration timing could not evolve (i.e., *h^2^* = 0). The rate of evolution was also greater, and positive effects on population persistence more pronounced, in simulations where the initial phenotypic variance was higher, which resulted in higher additive genetic variance (i.e., evolvability) for a given heritability and initial mean trait value, and therefore greater capacity to respond to directional selection. Although the absolute magnitudes of evolutionary and demographic responses were sensitive to the underlying functions relating migration survival to river temperatures and flows, the relative effects of evolution on extinction risk were robust in all scenarios explored (Fig. 6, Fig. S1).

The effects of evolution on persistence were greatest when rates of river warming were in the 2–3°C range (Fig. 5). For example, with a 2°C increase in mean river temperatures by 2100 the relative reduction in quasi-extinction risk for populations with a capacity for evolution (i.e., the drop in quasi-extinction risk compared to that faced by populations with *h^2^* = 0, expressed as a percentage of the latter) was 92% when *h^2^* = 0.75, 83% when *h^2^* = 0.5, and 47% when *h^2^* = 0.25 (Fig. 5). Current published estimates suggest that average summer water temperatures in the lower Fraser River might increase by 1–2°C by 2100 [19], [25], [26]. Two of these studies [19], [26] used output from GCMs based on the IPCC's emissions scenario A1B [46], which is considered a moderate climate change scenario, while the earlier predictions of [25], which used a slightly different methodology, were similar to those of [26]. However, should the growth of atmospheric carbon dioxide continue to accelerate as it has since the 1990s [49], regional air temperatures could rise at a faster pace than that predicted by these scenarios [50], and the current Fraser River models might therefore be conservative. Summer river temperatures at Hell's Gate increased at a rate of 0.3°C per decade between 1950 and 2006 [17], which if sustained would lead to a further increase of almost 3°C by 2100.

We do not draw conclusions regarding absolute risk of extinction for each climate change scenario explored in this study, given the considerable uncertainty inherent in the climate/hydrological scenarios and gaps in the understanding of the many ways in which climate affects salmon population dynamics [51]. Rather, our primary objective was to assess the difference that evolutionary adaptation *might* make to relative extinction risk, given a realistic set of demographic parameters and a well-characterized relationship between changing river temperatures and migration survival [19], [20], [38], [52]. The results suggest that evolution could make the biggest difference for future rates of river warming in the 1 to 3°C range (Fig. 5), which encompasses the spread of existing predictions [19], [25], [26]. Beyond that, extinction could be highly likely within 100 years regardless of evolution of migration timing, although concurrent physiological adaptation (e.g., improved cardiorespiratory performance at higher temperatures and increased aerobic scope) might allow some populations to keep pace with more extreme temperature increases [24].

Several lines of evidence suggest that the projected rates of phenology evolution necessary to ensure persistence within these bounds of river warming rates are entirely plausible. A recent review of quantitative genetic studies of salmonids found that phenological traits exhibited the highest heritabilities of any class of traits represented in the analysis, with a median heritability value of 0.51 [47]. Although heritability estimates are subject to a range of potential biases, and many of these estimates were made under experimental or captive-rearing settings [47], it highly likely that wild salmon populations harbor significant genetic variation for behavioral traits such as upriver migration timing. Consistent differences in run timing between geographically proximate populations experiencing different river thermal regimes strongly suggests genetically-based, climate-related divergence in this trait in the wild, although this might be more related to selection pressures at the spawning or egg incubation stages, rather than viability selection on migrating adults [53]. Contemporary evolutionary changes in migration timing were experimentally demonstrated to have occurred over 30 generations in Chinook salmon (*O. tshawytscha*) introduced to New Zealand [54]. Sockeye salmon in the Columbia River advanced their average river entry dates by about 6 days over 11 generations, coincident with a gradual increase in summer river temperature in recent decades [55], a response thought to be at least partly driven by natural selection [13]. Depending on the climate change scenario and underlying survival function used, migration timing advancements in our model were on the order of 7–14 days by 2100 for heritability values of 0.5, which translates to approximately 0.045 to 0.09 haldanes (PSDs per generation, assuming a PSD of 1 week and a generation time of 4 years). This compares with a median haldane value of 0.035 reported by [56] for studies of contemporary evolution over fewer than 80 generations across a range of species, and is also within the theoretical limits of sustainable rates of microevolution [32], [33].

One limitation of the current study is that we did not consider the possibility for adaptive phenotypic plasticity. Recent evidence suggests that many examples of purported microevolution related to climate change and other anthropogenic disturbances may in fact simply reflect plastic phenotypic changes [57], [58]. While we did model random inter-annual environmental influences on migration timing, we did not allow for individual (or indeed, genetically-based) variation in plastic responses, nor did we model potential correlations between cues affecting migration timing and river conditions influencing migration survival. Reliable environmental cues, if historically present, could have selected for adaptive phenotypic plasticity in migration timing, which could buffer the negative fitness consequences of future climate change for individuals and the population as a whole, to some degree [59]. Although weak correlations between oceanic variables and river entry dates have been documented for sockeye salmon in the Fraser River [60] and elsewhere, these are unlikely to reflect adaptive plasticity, as returning adults have limited opportunity to assess river conditions from the ocean given migration is initiated hundreds of kilometers from the river mouth [30]. ‘Last minute’ adjustments to migration timing based on more local (e.g., estuarine) cues might still occur, although these could incur fitness costs in addition to benefits. Oddly, some sockeye salmon populations that previously entered the Fraser River in early fall have recently started migrating 3–6 weeks earlier, exposing them to much higher water temperatures [61]. The reasons for this abnormal behavior remain unclear [41], [62], but the early migration phenomenon has resulted in extremely high (60–95%) in-river mortality in some years [61], [63], likely imposing strong selection for later migration [19]. Maladaptive or suboptimal phenotypic plasticity, whatever its causes, might play just as important a role in driving evolutionary and demographic responses in a changing climate as might adaptive plasticity, and further work is required to identify the environmental cues and constraints affecting migration timing plasticity in Pacific salmon.

For many species, microevolutionary responses will be essential for persistence in a warming world as current plasticity patterns will likely not remain optimal for long [9]. Limited genetic variation for traits subject to climate-related selection, however, will reduce the likelihood of evolutionary rescue; for example, genetic constraints on the rate of thermal adaptation have already been implicated in widespread extinctions of lizard populations experiencing climate warming [64]. This places a premium on management and conservation efforts that seek to preserve phenotypic and genetic variability, as a means to build insurance against ongoing climate change [65]. In the case of salmon, hatchery supplementation and captive breeding programs can alter the genetic composition and potential fitness of wild stocks [34], while strongly selective fisheries [35] might counter or swamp climate-induced selection pressures, potentially limiting the capacity of populations to keep evolutionary pace with changes in climate. Indeed, our simulations for Early Stuart sockeye salmon show that reduced phenotypic variance (and therefore reduced additive genetic variance) in migration timing results in weaker evolutionary responses for a given rate of river warming, which translates to an increased relative risk of quasi-extinction (Fig. 7). Interestingly, the risk of quasi-extinction was also slightly higher when phenotypic variance was lower but heritability was 0, indicating benefits of phenotypic diversity over and above those afforded by any increased capacity for evolution. The fitness function was asymmetric (sigmoidal), so the average lag load (i.e., the reduction in mean fitness resulting from a mismatch between optimal phenotypes and actual phenotypes) [32] was higher in populations with lower among-individual variance in migration dates, as the frequency of early-migrating (i.e., higher fitness) phenotypes was lower.

In our model, we assumed that all selection acting on upriver migration timing resulted from mortality induced by high temperatures or flows during the spawning migration, and that early migrating fish also spawned earlier. In reality, trade-offs (for example, between earlier migration and the potential need to spawn at a given date each year or to reach a certain size before leaving the ocean) might constrain the evolution of earlier upriver migration [13]. Adults might require higher energy reserves to survive the increasingly costly migration (during which they do not feed), but by leaving the ocean earlier in summer they potentially forgo some of the best growing opportunities [27]. Potential genetic covariances between migration timing and other heritable traits not considered in the model (e.g., spawn timing, which could be selected in a different direction to upriver migration timing in a changing climate) could also constrain evolutionary responses, although net evolutionary responses would be enhanced if the traits were selected in the same direction and are positively genetically correlated [13]. Although a univariate perspective on evolutionary response to climate change is certainly simplistic [66], a dearth of estimates of genetic correlations among fitness-related traits in wild salmon populations [47] limits meaningful assessment of constraints on multivariate phenotypic evolution. Plausibly, a single-trait approach provides a conservative analysis of potential evolutionary rescue, given that evolutionary changes in multiple characters could have a greater positive effect on mean fitness than the summed expected effects of changes in single traits [67]. For example, physiological adaptation in parallel with phenological adaptation could have synergistic positive effects on overall resilience, although debate continues among salmon biologists as to which is more likely during the upriver migration phase [13], [24]. On the other hand, the demographic benefits of adaptive responses at this phase of the life history might be offset by accumulating negative impacts of climate change across other life stages, which might reduce the overall productivity of stocks [68]. Strong selection sustained over many generations could also eventually erode genetic variance, thereby constraining future evolutionary potential, while reductions in population size could result in temporary bottlenecks in genetic variance that further reduce population viability [33]. We assumed constant genetic variance within families in our model (although between-family variance could change over time due to genetic drift or selection) and no linkage disequilibrium. These assumptions are probably reasonable for the timescales of selection considered, i.e., <25 generations [33], although future simulations could explore the consequences of relaxing them. We also assumed a constant age structure and non-overlapping generations for simplicity, which provided a reasonable approximation for this stock, given that 90% of returning adults spent one year rearing in freshwater, and two in the ocean [36]. Age structure might not remain constant in the future, however, and subtle changes could have important consequences for the eco-evolutionary dynamics, which could be explored with more complex versions of the model. The model could also be applied to other Fraser River sockeye salmon stocks, which differ from Early Stuarts in their age structure, intrinsic sensitivity to higher temperatures [24] or changing flow patterns, and the direction in which migration timing might be selected [19].

In summary, our results provide insights into how potential adaptation of migration timing might affect future persistence of salmon populations in a warming world. Losses in Fraser River sockeye salmon would have important consequences for the economy and culture of this region, as well as for the health of freshwater and marine ecosystems, but there remains considerable uncertainty about their future viability in a warming climate [51]. Predicting how populations might be affected by climate change remains a formidable but necessary challenge [64], [69]. In part, this reflects inherent uncertainties in scenarios of future greenhouse gas emissions, climate system responses, and difficulties pertaining to downscaling coarse-grained climate models to spatiotemporal scales relevant to population processes [46]. Still, the greatest impediment from a biological standpoint stems from limited mechanistic understanding of links between changing climate, organismal performance, and population dynamics and evolution. Our study illustrates the feasibility of integrating evolutionary processes, in addition to ecological processes, into models of population response to environmental change, which could improve our ability to effectively manage populations and conserve biodiversity in an uncertain and rapidly changing world.

## Supporting Information

Figure S1 **Sensitivity of main results to the shape of the temperature-survival function.** Sensitivity of evolutionary trajectories (left panels) and relationship between quasi-extinction risk and rate of river warming (right panels) to the *T_50_* and *b* parameters of the underlying sigmoidal survival-temperature function. Black curves: heritability of migration timing = 0; red curves: heritability = 0.25; green curves: heritability = 0.5; blue curves: heritability = 0.75. Evolutionary trajectory panels on the left show the change in mean migration timing in days (relative to the historic median Hell's Gate migration date of July 14) for a 2°C river warming scenario (i.e., a linear increase in mean river temperatures of 2°C by 2100). Panels A & B: *T_50_* = 18.8°C; *b* = 1.6. Panels C & D: *T_50_* = 20.8°C; *b* = 1.6. Panels E & F: *T_50_* = 19.8°C; *b* = 0.6. Panels G & H: *T_50_* = 19.8°C; *b* = 2.6.
(TIF)
