# Supplementary figures and images for: Time to Evolve? Potential Evolutionary Responses of Fraser River Sockeye Salmon to Climate Change and Effects on Persistence
Source: PLoS One. 2011 Jun 28;6(6):e20380. doi: 10.1371/journal.pone.0020380 (PMC3125159; doi:10.1371/journal.pone.0020380)

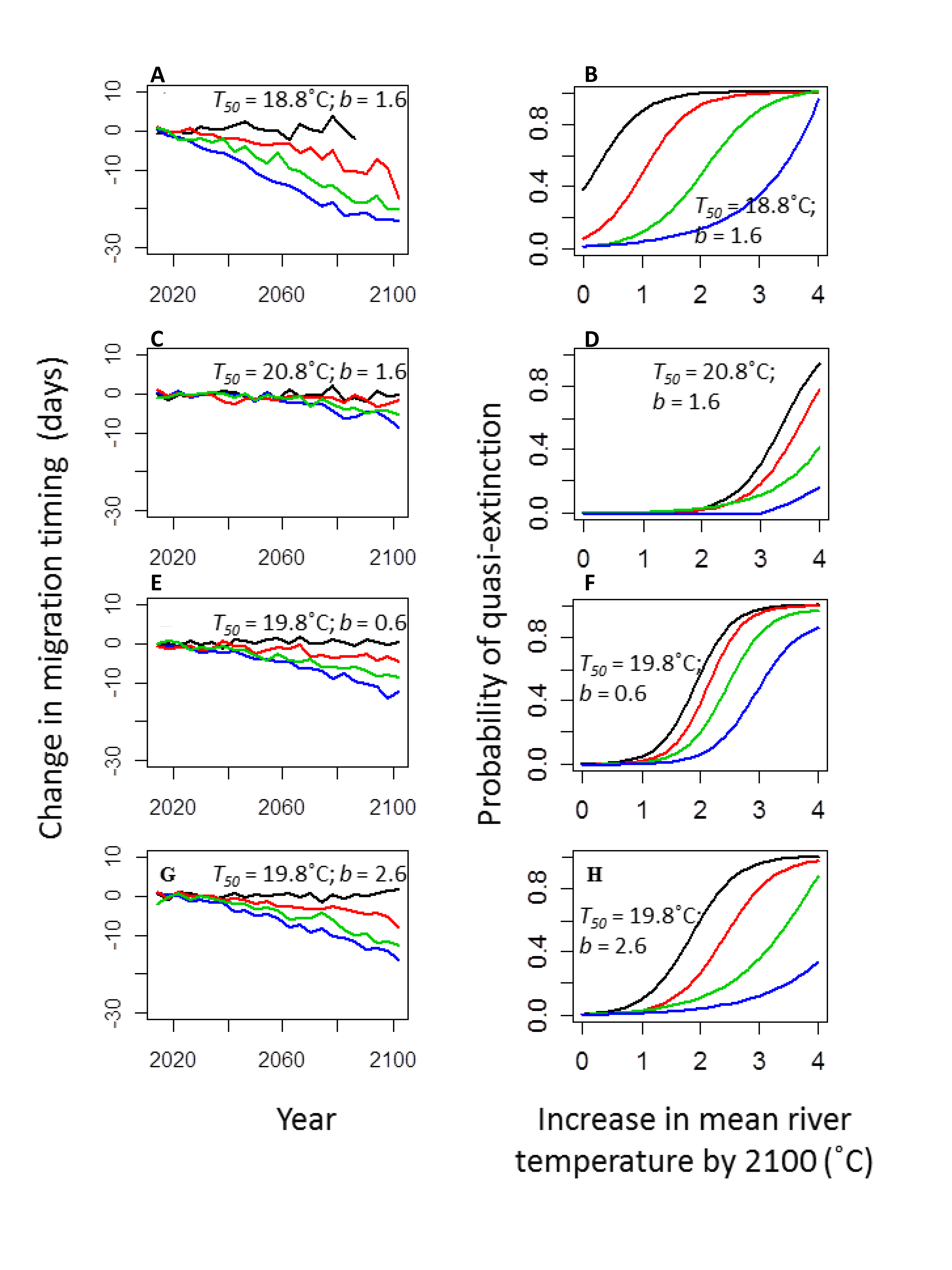

Supplement: Figure S1 — Sensitivity of main results to the shape of the temperature-survival function. Sensitivity of evolutionary trajectories (left panels) and relationship between quasi-extinction risk and rate of river warming (right panels) to the T50 and b parameters of the underlying sigmoidal survival-temperature function. Black curves: heritability of migration timing = 0; red curves: heritability = 0.25; green curves: heritability = 0.5; blue curves: heritability = 0.75. Evolutionary trajectory panels on the left show the change in mean migration timing in days (relative to the historic median Hell's Gate migration date of July 14) for a 2°C river warming scenario (i.e., a linear increase in mean river temperatures of 2°C by 2100). Panels A & B: T50 = 18.8°C; b = 1.6. Panels C & D: T50 = 20.8°C; b = 1.6. Panels E & F: T50 = 19.8°C; b = 0.6. Panels G & H: T50 = 19.8°C; b = 2.6. (TIF) [file pone.0020380.s001.tif]
